# Behavioral Patterns of Children Involved in Bullying Episodes

**DOI:** 10.3389/fpsyg.2018.00456

**Published:** 2018-04-10

**Authors:** Carlos V. Santoyo, Brenda G. Mendoza

**Affiliations:** ^1^Laboratorio de Desarrollo y Contexto del Comportamiento Social, División de Investigación y Posgrado, Universidad Nacional Autónoma de México, Delegación Coyoacán, Mexico; ^2^Facultad de Ciencias de la Conducta, Universidad Autónoma del Estado de México, Toluca, Mexico

**Keywords:** bullying, behavioral patterns, children, victims, teachers

## Abstract

This study applied a systematic observation strategy to identify coercive behavioral patterns in school environments. The aim was to describe stability and change in the behavioral patterns of children identified as victims of bullying. To this end, the following specific objectives were defined: (1) to identify episodes of bullying based on the frequency of negative behaviors received and power imbalances between bully and victim; (2) to describe stability and behavioral changes in student victims based on their social and academic conduct and the aggression they receive from peers and teachers; and (3) to describe the functional mechanisms responsible for the process of social organization (i.e., the Social Effectiveness, Social Responsiveness, and Social Reciprocity Indexes). The sample consisted of nine children identified as victims, nine classified as bullies, and nine matched controls, all elementary school students from the study developed at the National Autonomous University of Mexico files. A multidimensional/idiographic/follow-up observational design was used. Observational data describes asymmetry between victims and bullies based on microanalyses of the reciprocity of their behavioral exchanges. In addition, the behavioral patterns of victimized children were identified in relation to their academic activity and social relationships with peers. A model of coercive reciprocity accurately describes the asymmetry found among bullies, victims, and controls. A reduction in victimization was found to be related to: (1) responsiveness to the initiation of social interactions by peers and teachers; and (2) the time allocated to academic behavior during the study.

Studies of bullying have already identified serious detrimental effects, both short- and long-term, not only for victims (Lereya et al., 2015) but also for passive observers and the bullies themselves. Finally, it is clear that bullying negatively affects a school's social climate (Beaudoin and Roberge, 2015). Bullying is a type of aggression exhibited in a persistent manner via coercive behavior toward a person(s), with an existing power asymmetry between victim and aggressor (Olweus, 2001). Coercive behavior is expressed as the combination of functional events, bi-directional and generally asymmetric, where a person manipulates the conduct of others using the contingent presentation of aversive events that are removed when the others behavior takes the desired direction (Patterson, 1979).

In general, bullying has been observed using indirect measures (Olweus, 1993), as only a few studies 31 of 1,471 used observational methodologies that can provide novel empirical evidence for evaluating this phenomenon (Machado et al., 2015). Direct observation of patterns of social interaction facilitates identifying how social relationships are established, maintained, and modified (Cairns, 1979; Bakeman and Gottman, 1986; Espinosa, 2017), making this methodology suitable for the study of bullying. From an ecological perspective, this field takes into account the outcomes of how intra- and inter-individual dimensions relate over time (Modecki et al., 2014), based on multi-method research approaches.

Previous research on patterns of coercive behavior have used multi-method approaches that include behavioral data, self-reports and reports from peers and other adults, as well as sociometric indicators of behavior, status, and consensus (Santoyo et al., 2007b, 2008). The theoretical framework for this study corresponds to the sinthesys approach of Cairns (1979), and its implications to school settings (Cairns and Cairns, 1994), which is framed in the social organization processes, through which it is possible the study of social networks of the students through ecological social analysis, that has a sociometric technique called Social Cognitive Maps (SCM; Farmer and Cairns, 1991).

The analysis of social ecology in this perspective lies in studying the relevant elements of social links that children maintain at the school environment. SCMs, allow to identify the subgroups of students of a classroom, allowing to analyze their social interactions in the school setting.

For the analysis of the social links, it is necessary to use analyses of the *Functional Mechanisms* responsible for the process of social organization, and the use of the following social competency scales have been proposed: Social Effectiveness (SEI), Social Responsiveness (SRI), and Social Reciprocity Indexes (Santoyo, 1996).

As defined in Equation (1), the social effectiveness index describes the relative frequency of initiating acts by the target subject (TS) that result in a social episode (i.e., successful initiating acts), relative to the total number of initiating acts (with or without a peer response)

(1) $$SEI=\frac{Succesful\;initiating\;acts}{Total\;initiating\;acts}$$

As Equation (2) shows, the Social Responsiveness Index describes the relative frequency of successful initiating acts directed toward the target that result in a social episode, relative to the total number of social initiating acts directed toward the target (with or without a target response).

(2) $$SRI=\frac{Social\;response\;to\;acts}{Total\;initiating\;acts}$$

The third index reflects coercive reciprocity. It is useful for distinguishing behavioral symmetry between children who exhibit aggressive behavior and those who do not (Santoyo et al., 1996, 2007c; Espinosa, 2017). According to this index, aggressive children exhibit higher reciprocity in their coercive exchanges than a matched group (Santoyo et al., 2008, 2017). This kind of effect has also been found in the conflictive interaction of violent spouses (López and Santoyo, 2004): This relationship can be expressed using the coercive reciprocity model, as shown in Equation (3).

(3) $$\frac{Nbe}{Nbe+Nbr}=\frac{Nsep}{Nsep+Nser}$$

The first part of this equation represents provocation events, where Nbe *(Negative behavior emitted)* indicates physical or verbal coercive behavior from the target toward a peer without the peer having addressed the target during the interval immediately preceding such behavior. Nbr *(Negative behavior received)* represents physical or verbal coercive behavior directed toward the target without provocation by her/him during the interval immediately preceding such behavior. The second part corresponds to the consequences of provocation: *Nsep (Negative social episodes produced)* represents negative social episodes initiated by the target, while *Nser (Negative social episodes received)* shows negative social episodes initiated by a peer. Negative social episodes are defined as physical and/or verbal behaviors between the focal subject and other persons that occur either simultaneously or successively, with mutual dependence on the participants' behavior.

Our review of current literature found no previous studies that attempted to identify these kinds of functional mechanisms of bullying based on dyadic interactions and assessments of mechanisms of effectiveness, responsiveness, and reciprocity.

In this study, the power imbalance or asymmetry between victim and bully was identified by analyzing the reciprocity mechanism (Equation 3) in light of the definition by Atlas and Pepler (1998), in which victims are defined as students who are targets of negative behaviors, and bullies as those who frequently initiate such behaviors. We adopted a synthesis approach based on implementing social ecology analysis (Cairns, 1979) that allows a better understanding of how both individual and social behavior among the members of a social network are regulated. This analysis is appropriate because studies have consistently shown that a risk factor for victimization by bullying is a lack of social links in the school environment (Salmivalli et al., 1996; Eslea et al., 2003; Mendoza and Maldonado, 2017). The present study provides evidence of social links based on the use of SCM (Farmer and Cairns, 1991), which could overcome the limitations of conventional sociometry, such as restricting nominations to only a few people, or naming a certain number of classmates when in reality no sustained relationship exists among them. The use of SCMs has the additional advantage of allowing the identification of existing sub-groups supported by statistical criteria.

Our study thus proposes an approach to the study of behavioral patterns exhibited by victims of bullying that employs an observational methodology in school settings.

This research is thus an extension of Study developed in school environments with a 3-year follow-up period (Santoyo, 2007; Santoyo and Colmenares, 2012), data are used to estimate densities of individual and social activities, construct behavioral profiles of individuals, identify types and frequencies of social interactions, and integrate information on contextual and dyadic exchange. Thus, it is consistent with previous methodological strategies based on observing interactions and studies of social development in natural settings (Cairns et al., 1991).

Finally, our goal is to describe the behavioral patterns of children identified as victims of bullying in their social ecology. Achieving this entails addressing the following specific objectives: (1) identifying episodes of bullying based on the frequency of negative behaviors received and power imbalances between bully and victim; (2) describing stability and behavioral changes in student victims based on their social and academic conduct and the aggression they receive from peers and teachers; and (3) describing the functional mechanisms responsible for the process of social organization using the Social Effectiveness, Social Responsiveness, and Social Reciprocity Indexes. For this purpose, children in different grades of elementary school were selected. The study focuses on the behavioral and dyadic patterns of victims of bullying, matched controls, and bullies. Finally, a microanalysis of behavioral patterns was performed to obtain information on the conditional probabilities of behavioral acts by participants.

## Methods

### Participants

The sample included 27 elementary school students aged six to nine. All subjects were attending a public school in Mexico City (first to third grade). The average number of children per classroom was 28. Nine of the children were identified as victims, nine others were matched as bullies, and nine were selected as matched controls. Written authorization to perform the research was obtained from school authorities. The project was approved previously by an ethics committee at the lead author's university. This work used behavioral observation methods which carefully preserved the identity of children and teachers. Observers never stood closer than 10 m from the children or interacted with them during the sessions. It should be pointed out that, at the time, observational records and notes were not considered as potentially damaging or harmful, given that they would never be made public or include personal identification of participants.

### Design

A multidimensional/idiographic/follow-up observational design (Blanco-Villaseñor et al., 2003) was applied.

### Selection criteria

#### Victims group

To be eligible to participate in the focal group, a student had to fulfill the following criteria:

1. Belong to the highest quartile for the frequency of negative behavior received, relative to the absolute frequency of positive and coercive behavior emitted by their peers.
2. Belong to the highest quartile for the absolute frequency of negative behavior received.
3. Coercive behavior emitted by the target could not exceed 6% of the student's total social behavior.

Only nine children were identified as victims. Then, other 18 children were selected as a member for each matched comparison group (control and bullies group).

#### Matched control group

For comparative purposes, for each child identified as a victim based on the selection criteria, a matched peer with similar characteristics of gender, age, group, and school grade was selected.

#### Bullies group

In order to analyze the asymmetry between victims and bullies, and for purposes of comparison, nine students identified as bullies were included after verifying the following requirements:

1. Belonging to the highest quartile for the frequency of negative behavior emitted; and
2. Having coercive behavior that exceeded 6% of their total social behavior. This criterion identifies children that exhibit aggressive behavior, criterion established by Patterson (1982) and more recently supported and extended by empirical evidence by Cruz (2007) and Santoyo and Colmenares (2012) in school settings.

#### Setting

All observations took place in typical classrooms (during lessons) at a public elementary school in the south of Mexico City. The classrooms had adequate lighting and ventilation.

#### Instruments

Participants' behavioral data were collected based on the *Observational and Behavioral System of Social Interaction (OBSSI)* (Santoyo et al., 1994), which was designed specifically to study social interaction in school settings. The OBSSI makes it possible to identify events and situations that constitute behavioral patterns. It is an exclusive, exhaustive behavioral categories system based on 5-s intervals and constituted by representative behavioral categories for the actions that participants (previously designated as “target subjects”) exhibit in educational settings. These categories are organized as follows:

- Individual behavior: on-task, off-task, individual play
- Social interaction:
  - Social actions initiated by a target child and directed at another person;
  - Social actions initiated by others and directed at a target child;
  - Such dyadic and group social interactions as: coercive behavior, group play, sharing, conversation, physical contact, etc.
- Other responses:
  - Behavior displayed by target child that is not covered by the other categories in the *Observational and Behavioral System of Social Interaction*.

The *Observational and Behavioral System of Social Interaction* thus generates an event-based, sequential record in which observers write the order of occurrence of events. Moreover, it allows the study of contextual factors at the site where a behavioral pattern emerges (i.e., classroom, playground, math lessons, Spanish lessons, etc.), and identifies the person who initiates an exchange. The use of this system made it possible to categorize participants' activities, the quality of social exchanges (coercive or prosocial), the social agents involved in social interactions (peers or teacher), and the direction and location of exchanges. Meanwhile, the contents of specific actions involving children are described by keywords or verbs that express the type of action emitted. For this study, the *Observational and Behavioral System of Social Interaction* categories used were: *Initiating acts, Response to acts, Social Interaction (identified as positive or negative), Academic activity (on-task behavior)* and *Other responses (off-task behavior)*.

Using *Observational and Behavioral System of Social Interaction*, researchers can identify three types of aggressive behavior: physical, verbal, and coercive or negative. Obviously non-aggressive behavior is also recorded. Previous studies obtained a 0.95 generalizability coefficient (Espinosa et al., 2006). This value indicates that the results from individuals and sessions can be reliably generalized based on the category system, number of participants, and the number of sessions programmed.

To describe social ecology, the sociometric technique called SCMs (Farmer and Cairns, 1991) adapted for use in Mexico was used (Santoyo and Espinosa, 2005). Here, students from the same class as the focal subjects were asked, individually, two questions: “Are there people in the class who hang around together a lot?” and “Are there people in the class who do not have a group?” When the interviewee did not include him/herself in any group, we asked: “What about you; do you have a group you hang around with at school? Based on these interviews, we generated a complete social network that identified the structure of relationships in the form of groups, sub-groups and isolated children. To identify social links and groups a co-occurrence matrices were designed, where each child was listed on the horizontal axis as a respondent and on the vertical axis as a nominee. The inclusion of a child in a sub-group was determined by a correlation equal to, or higher than, 0.40 (Farmer and Cairns, 1991). We classified weak and strong connections in the social network as those that showed significant correlations at the level of 0.01 and 0.005, respectively.

### Procedure

A total of 84 children were observed during normal classroom lessons. The criteria applied identified nine children as victims, nine as bullies, and nine as matched controls. The SDIS-GSEQ program (Bakeman and Quera, 2011) was used to identify behavioral patterns (Santoyo et al., 2006). Each child was observed in focal samples for 90 min per year for 3 years.

Behavioral field data were collected using the *OBSSI*, followed by information from self-descriptive behavior and teachers' descriptions of the students in the class. This procedure was employed for 3 years, the data (cohorts) is derived from a study developed at the National Autonomous University of Mexico called Coyoacán Longitudinal Study, and allowed us to compare data stability throughout the follow-up period, as well as the behavioral patterns of participants in the three groups (victims, bullies, and controls). The sample was obtained on a *post hoc* basis (Elder et al., 1993), and during data collection neither the researchers nor the observers knew the status that had been assigned to the children (i.e., victims, bullies, or matched controls). In general, this strategy is based on a person-oriented approach (Cairns et al., 1998).

Pairs of trained observers collected the behavioral data, which satisfied the criterion of 80% reliability. In addition, a sample of the records (65%) was obtained and the Cohen's Kappa index (Cohen, 1960) was calculated, obtaining indexes of 0.81 for target subjects and 0.89 for matched controls. According to the parameters established by Fleiss (1981) and Bakeman and Gottman (1986), these data indicate excellent concordance.

#### Behavioral sampling and interviews

In order to compare behavioral patterns, the time allocated to different activities, and participants' social and behavioral preferences, a behavioral sampling was carried out *in situ* based on parameters tested in earlier studies (Santoyo et al., 2007b). For each participant, sampling entailed six 15-min sessions during classroom lessons. Efforts were made to conduct observation of each participant on consecutive days. Each subject was observed in the classroom for 90 min. To avoid interfering with the quality of the behavioral data, the interviews held to describe the social ecology, SCMs were implemented 1 week after completing the behavioral sampling. In this case, each student from the same class as the victim, bully and matched child was interviewed independently until the entire sample had been seen (*n* = 84).

Comparisons of the status and wave of measurement occasion were performed (Status = 3: victim, bully, matched control group; wave measurement occasions = 3: 1, 2, and 3). The sample was obtained on a *post hoc* basis, and during data collection neither the researchers nor the observers knew the status that had been assigned to the children—i.e., victims, bullies, or matched controls in a natural setting.

## Results

### Characteristics of the bullying episodes

The first part of our results identifies the characteristics of the bullying episodes with the profiles of the students classified as bullies or victims. The outcomes of the power imbalance derive from one of the aforementioned equations of the *functional mechanisms* responsible for social interactions (Ec. 3, as outlined in the Introduction). One of the criteria used to distinguish bullying was the *frequency of negative behaviors received*. Student victims received negative behaviors more frequently than bullies or the matched control children. On average, victims received 13, 5, and 1%, respectively, of negative behavior from their peers in the first, second and third waves of measurement. The latter figure represents a significant decrease, as shown by the results of a Tukey's test (first, 0.13 ± 0.01; second, 0.05 ± 0.01; third, 0.01 ± 0.008).

A Tukey's multiple comparison test also showed that victims received more negative behavior (0.10 ± 0.009) during the first wave of measurement than the bullies (0.02 ± 0.017; 0.02 ± 0.01; 0.009 ± 0.008) and the matched controls during the first, second and third waves of measurement (0.01 ± 0.17;0.02 ± 0.01;0.017 ± 0.008, respectively), [*F*_(4, 42d.f.)_ = 5.67, *p* < 0.001].

**Figure 1:**
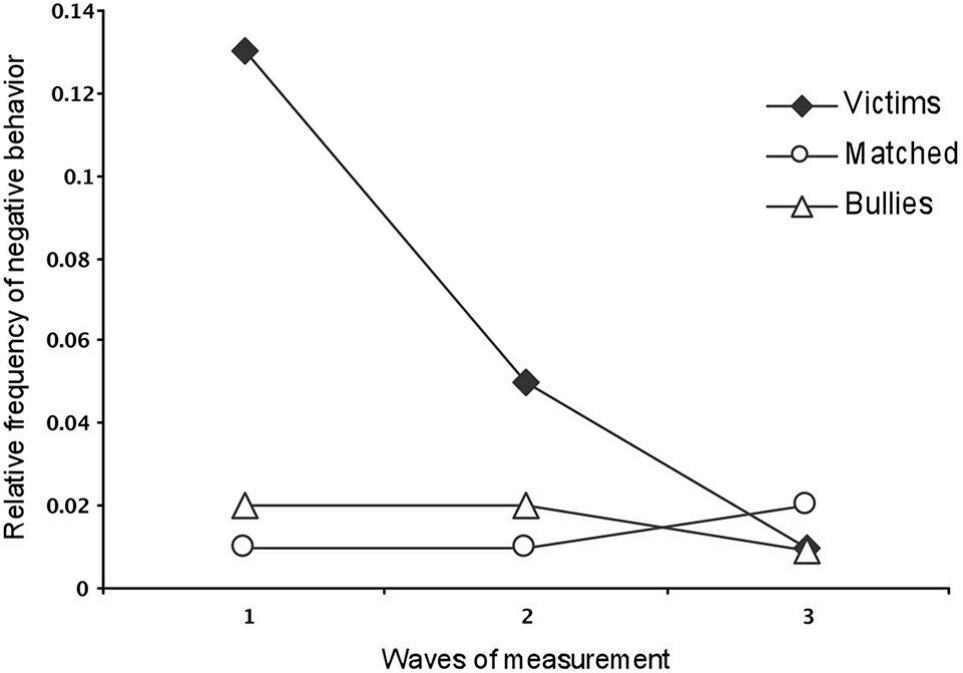
Relative frequency of negative behaviors that peers directed to students in the victim, bullies, and matched-Control groups during the first waves of measurement.

Figure 1 shows the frequency of negative events received by the victims, bullies, and matched controls. The victims received a higher frequency (0.06 ± 0.006) of negative behaviors than bullies (0.02 ± 0.006) and controls (0.01 ± 0.006), and were the targets of over 5% of negative behaviors out of the total number of positive and negative behaviors directed at them [*F*_(2, 21d.f.)_ = 17.81, *p* < 0.001].

The effect size was calculated, and a value of the effect size *f* = 0.05 was obtained, showing an effect size with a mean value (Cárdenas and Arancibia, 2014).

Finally, results show that in the third wave of measurement, 78% of the victims group exhibited a reduction in the relative frequency of harassment received, relative to the first wave (Figure 1).

**Figure 2:**
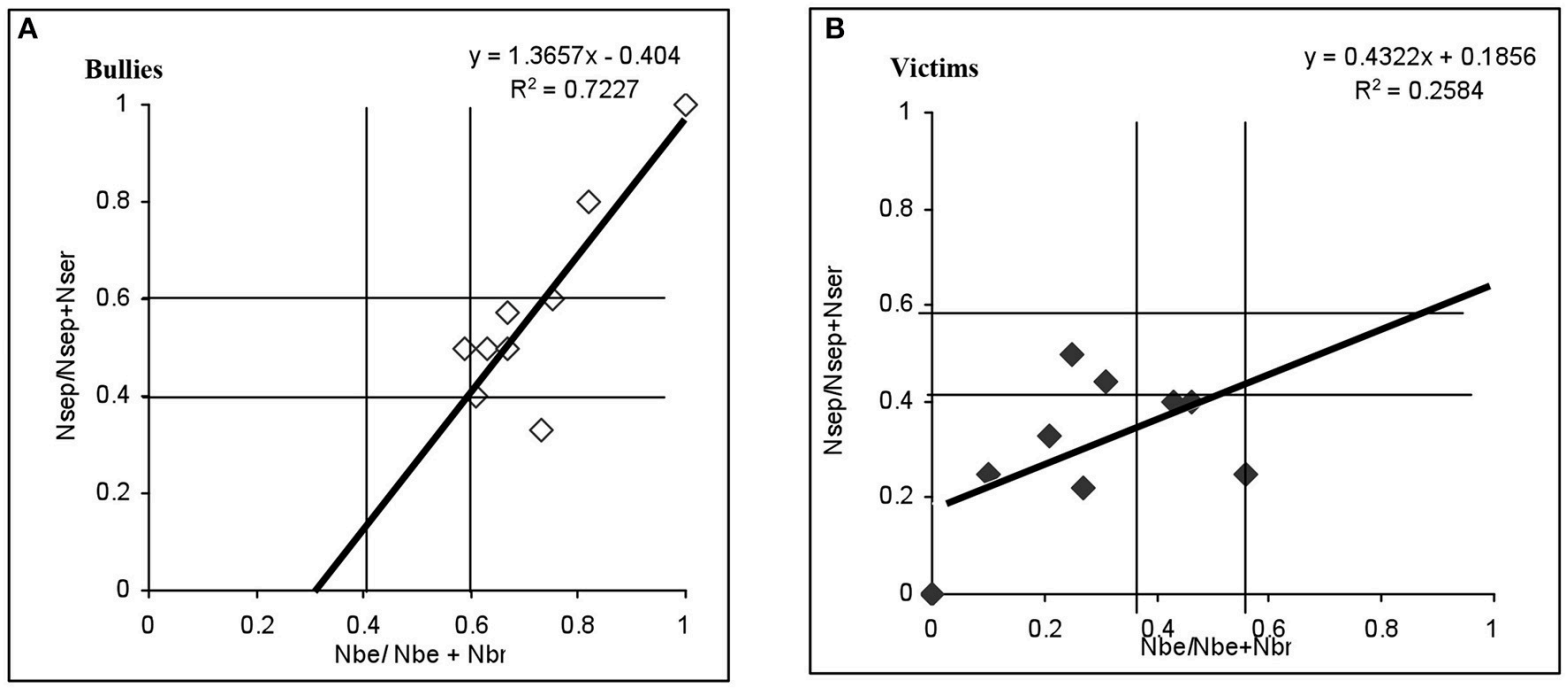
Reciprocity of coercive events in children identified as bullies **(A)** and victims **(B)**, from the three school grades, during the first wave of measurement (from Equation 3).

Another criterion that distinguished bullying was the power imbalance, or asymmetry, between victim and bully. This relation was evaluated with the coercion reciprocity index derived from Equation (3). The reciprocity index was obtained for 100% of victims, bullies and controls. Figures 2A,B show the analysis of the reciprocity of coercive episodes for victims and bullies. The abscissa axis corresponds to relative provocations, the ordinate axis to their relative consequences. Values of 0.40–0.60 indicate high symmetry in provocation frequency; values above 0.60 indicate that the bullies consistently provoked conflicts; while values below 0.40 indicate that the conflicts were initiated by peers (Santoyo et al., 2007b).

Figure 2A, shows that most of the bullies (six out of nine) consistently instigated conflicts (values above 0.60). The regression analysis based on Ec. 3 yielded an *r*^2^ = 0.72, which confirms the bullies' coercive reciprocity.

The size of the effect was calculated and a value of the effect size *f* = 2.5 was obtained, showing an effect size with value denominated high (Cárdenas and Arancibia, 2014).

Figure 2B, shows that victims (eight out of nine) exhibited values below 0.60; that is, they were targets of coercive behaviors without provoking conflicts. The weak value of *r*^2^ = 0.25 indicates that they tended not to respond symmetrically to the coercive behavior they received.

In summary, the power imbalance or asymmetry between bullies and victims was confirmed by the difference in the *r*^2^-values and by the asymmetry in the location of the values obtained for these two groups based on the model of negative reciprocity (Ec. 3).

### Stability and change behavior: victims

**Figure 3:**
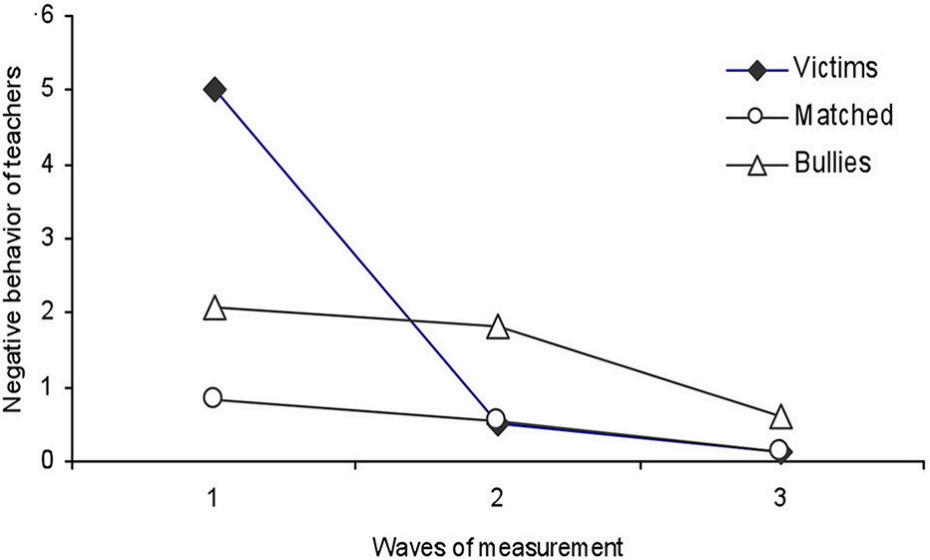
This figure shows the behavior negative frequency that teachers directed to students in the victim bullies and matched-Control group, during the entire sampling period.

The second part of our results—shown below—describes the stability and change of behavior patterns identified for the victims, including social outcomes, academic behavior and aggression from teachers. The most striking result of Figure 3 is that in the first wave of measurement the children in the victims group received a higher average frequency of negative behaviors from teachers (5 ± 0.99) than bullies (first, 2 ± 0.91; second, 1.8 ± 0.38; third,0.62 ± 0.27) and the matched controls (first, 0.82 ± 0.99; second, 0.55 ± 0.38; third, 0.12 ± 0.27). These results were confirmed by a Tukey's multiple comparison test [*F*_(4, 42 d.f.)_ = 4.85, *p* < 0.01].

The effect size was calculated, and a value of the effect size *f* = 0.07 was obtained, showing an effect size with medium value (Cárdenas and Arancibia, 2014).

We also observed a reduction in the average frequency of negative behaviors received by victims across the waves of measurement (5 ± 0.99; 0.50 ± 0.38; 0.12 ± 0.27, respectively, for the first, second and third waves), a finding consistent with the decrease in harassment that peers directed at victims.

**Figure 4:**
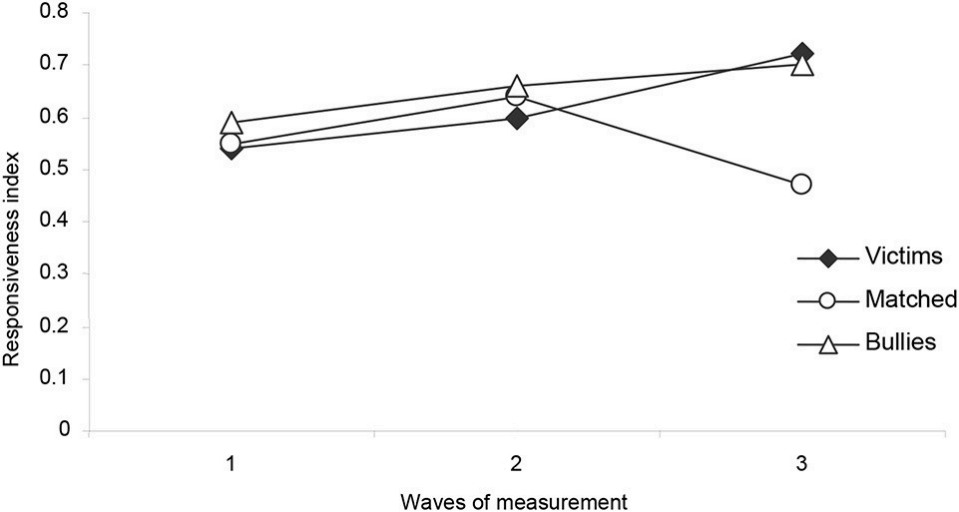
Average relative frequency of victim, bullies and matched-Control children's responses to social initiatives from peers (from Equation 2) during the three waves of measurement.

Shown below are the results derived from the *functional mechanisms* responsible for the process of social preferences. With respect to the pattern of social behavior manifested by the student victims, Figure 4 shows that they exhibited a greater increase in the value corresponding to the Social Responsiveness Mechanism (Equation 2) in the transition from the first (0.54 ± 0.03) to the third wave of measurement (0.72 ± 0.07). In the third wave, the matched control children established positive interactions with their peers at a rate of 47%; in contrast, the children in the victims group responded to 72% of their peers' attempt to initiate social interactions, and were able to establish positive interactions with them. This greater increase in the value corresponding to the social responsiveness index by the victims group is consistent with the decrease in negative behavior that victims received from their peers. These results were also confirmed by a Tukey's multiple comparisons test [*F*_(4, 42d.f.)_ = 3.62, *p* < 0.05].

The effect size was calculated, and a value of the effect size *f* = 0.07 was obtained, showing an effect size with medium value (Cárdenas and Arancibia, 2014).

**Figure 5:**
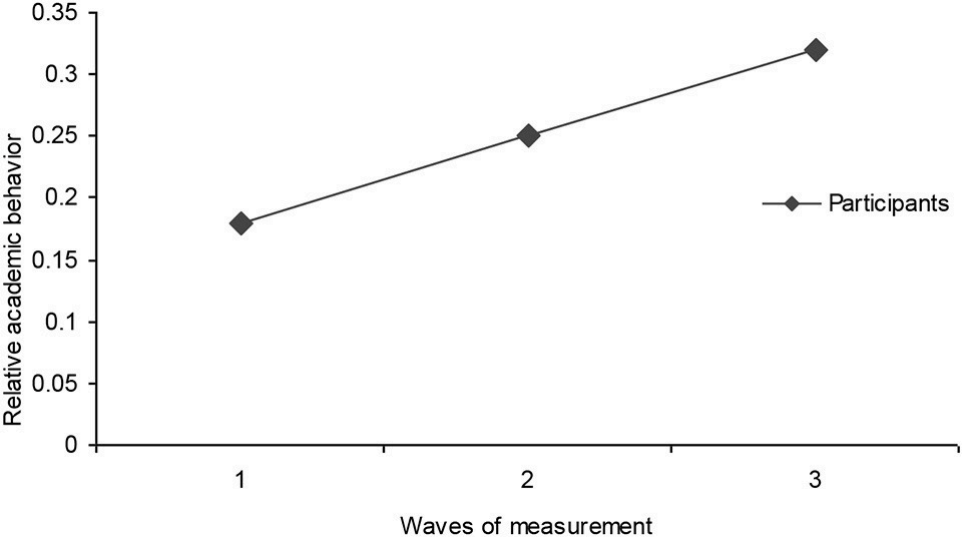
This figure shows the average time (in seconds) that students (victim, bullies, and matched-Control) allocate to academic behavior relative to the time spent on other behaviors during the three waves of measurement.

Figure 5 shows that students from all three groups allocated 18% of their time to academic behavior during the first wave of measurement (0.18 ± 0.01), and that this increased to 25% in the second (0.25 ± 0.02), and 32% (0.32 ± 0.02) in the third. Hence, the amount of time assigned to academic work almost doubled in the transition from the first to the third waves. These results were confirmed by a Tukey's test [*F*_(2, 42d.f.)_ = 11.14, *p* < 0.001].

The effect size was calculated, and a value of the effect size *f* = 0.07 was obtained, showing an effect size with medium value (Cárdenas and Arancibia, 2014).

### Social cognitive maps (social ecology)

The description of the social ecology of the study setting is based on the SCMs of children in grades one two and three during the first wave of measurement (see Table 1). We found that the matched control group had more links (44) than the children in the bullies (39) and victims groups (34). It is important to point out that two victims exhibited no links to their peers, while 77% of the children identified as bullies and matched controls, as well as 66% of the children identified as victims, had more than three links with their peers.

**Table 1:** Total number of connections in the SCM by school grade as a function of the group to which students belong (victim, bully, and matched group); weak and strong connections (0.01 and 0.005 correlation coefficients, respectively).

**Groups**	**Victims**		**Bullies**		**Matched**		**Total**	
**Level of Social Network**	**Low**	**High**	**Low**	**High**	**Low**	**High**	**Low**	**High**
Links between peers	27	7	27	12	31	13	85	32
Sub total	34		39		44		117	

The negative behaviors of peers directed at the target children, and the corresponding responses of those children (bullies, victims and matched controls) were also examined (see Table 2), together with the target children's negative behavior directed toward others and the corresponding immediate responses of their peers (see Table 3). This analysis allowed us to identify whether or not the victims, bullies and matched control students became involved in negative social interactions in response to another classmate's negative behavior, or when they directed coercive behavior toward others. Results indicate that once victims received a coercive event, their probability of responding was 0.46 (classified as “inhibitory” with an adjusted residual value of −2.5), in contrast to the matched controls whose probability of responding to a provocation was 0.74 (classified as “excitatory” with an adjusted residual value of 1.9). These results were confirmed by an *X*^2^-value of (2 d.f.) = 7.77 (*p* < 0.05) (see Table 2).

**Table 2:** Conditional probability analysis of episodes of negative behavior received by SF (Nbr).

	**Negative social interactions resulted**		
**Events of negative behavior received by target *(Nbr)***	***Nser* (ON)**	***No Nser* (OFF)**	**Total**
**VICTIMS GROUP**			
Frequency	41	47	88
Conditional probability	0.47	0.53	
Adjusted residual	−2.5^*^	2.5^*^	
**BULLIES GROUP**			
Frequency	31	15	46
Conditional probability	0.67	0.33	
Adjusted residual	1.7	−1.7	
**MATCHED GROUP**			
Frequency	20	7	27
Conditional probability	0.74	0.26	
Adjusted residual	1.9^*^	−1.9^*^	

FS getting involved (ON) or not getting involved (OFF), in negative interactions when they are the target of a provocation.

* *p < 0.05*.

**Table 3:** Conditional probability analysis of episodes of negative behavior emitted by SF (Nbe).

	**Negative social interactions resulted**		
**Events of negative behavior emitted by Target (Nbe)**	***Nsep* (ON)**	***No Nsep* (OFF)**	**Total**
**VICTIMS GROUP**			
Frequency	16	15	31
Conditional probability	0.52	0.48	
Adjusted residual	2.2^*^	−2.2^*^	
**BULLIES GROUP**			
Frequency	46	113	159
Conditional probability	0.29	0.71	
Adjusted residual	−2.7^*^	2.7^*^	
**MATCHED GROUP**			
Frequency	16	20	36
Conditional probability	0.44	0.56	
Adjusted residual	3.6^*^	3.6^*^	

*FS getting involved (ON) or not getting involved (OFF), in negative interactions when FS emitted negative behavior to others*.

* *p < 0.05*.

The effect size was calculated and a value of the effect size was obtained *f* = 0.5, showing an effect size with value denominated high (Cárdenas and Arancibia, 2014).

To extend the analysis, Table 3 presents the results from the consequences of negative behavior emitted by the target children. This shows that the bullies group had a probability of 0.29 of receiving negative behavior when they directed physical or verbal coercive behavior toward other peers (inhibitory with an adjusted residual value of −2.7, in contrast to the victims group, whose probability of receiving negative behavior was 0.52; that is, excitatory with an adjusted residual value of 2.2 [*X*^2^_(2 d.f.)_ = 6.99 (*p* < 0.05); see Table 3]. The effect size was calculated and a value of the effect size was obtained *f* = 0.45, showing an effect size with value denominated high (Cárdenas and Arancibia, 2014).

## Discussion

### Characteristics of the bullying episodes

In order to comply with the general objective of the study, we first employed systematic observation to analyze whether the aggressive events fulfilled the characteristics of bullying. In this case, the frequency of negative behavior directed toward victims, which has traditionally been measured using indirect instruments (Olweus, 1993).

Systematic observation used herein allowed us to overcome some of the difficulties associated with studying bullying at early ages, such as the distinction between aggressive behavior and bullying, identifying power asymmetry, and the persistence of negative behavior directed toward victims (Machado et al., 2015).

### Stability and change behavior: victims

Based on study data, we propose that in order for a child to be identified as a victim, 5% or more of the behavior that she/he receives from peers must be negative. This proposal extends the criterion suggested by Santoyo et al. (2007b) for identifying the coercive behavior of aggressive children with a sample of victims. Most social behavior is positive and some level of aggressive behavior is sometimes expected. In antisocial adolescents population (Patterson, 1982) risk boys shows more than 5% or more of negative behavior and control boys shows less than such percentage; for that, this criterion was proposed for student victims which are consistent with the adaptation proposed by Cruz (2007) with Mexican children in school settings. Our study further demonstrates that bullying is frequent in the school environment, a finding consistent with the results of Elgar et al. (2015). Thus, bullying is not necessarily “covert” (Olweus, 1993) or uncommon in the presence of adults (Landau and Swerdlik, 2005). Therefore, the systematic observation strategy proposed made it possible to identify the behavioral patterns of victims, bullies and matched children in school environments.

With respect to the general objective of the study, we were able to identify through our results that the behavior pattern of the children identified as victims evolved until they stopped “being so” (in the third cohort). This suggests that being able to cease being victimized is not related simply to the acquisition of social abilities but, rather, depends on several developmental and regulatory factors that do not develop in isolation, but appear to protect students who are at risk of being bullied. These results differ from those of Whitney and Smith (1993) and O'Moore et al. (1997), who indicate that the frequency of victimization remains constant in students aged seven to nine, decreases during the transition from elementary to middle school, and falls to zero when children reach the age of 16. It should be noted, however, that the sample in our study includes only a 3-year follow up, during elementary school education. Additional longitudinal studies are suggested which extend such follow up period.

These results suggest that a relationship exists between negative behavior that victims receive from their peers and from their teachers, findings that strengthens the evidence in studies developed with verbal reports, which indicate that victims of bullying could be also victims of their teachers (Mendoza, 2011, 2013). Next studies must search to the quality of social episodes with teachers. Also, this result is highly consistent with Wilson and Herrnstein (1985) theory about crime and human nature which highlights the role of strengthening non-aggressive behavior as a way of strengthening incompatible patterns of response with bullying and victimization, like prosocial behavior or academic behavior.

With regard to the objective of the study, and descriptions of the changes in the behavior patterns of child victims, our results suggest that the reduction in victimization is related to being highly responsive to the social initiatives of one's peers (i.e., a high social responsiveness index) and to academic motivation. Indeed, consistent with matching law (Wilson and Herrnstein, 1985), the groups of children (victims, bullies, and matched controls) became integrated, and a clear reduction in victimization was shown as the time assigned to academic behavior increased. These results represent a social and motivational index of great impact that may be related to decreased victimization in the classroom. This evidence also supports the findings of Turunen et al. (2017), who demonstrated that bullying interferes with both the victims' and the bullies' learning, coupled with the observation that coercive children show a low preference for academic activities (Cuenca and Mendoza, 2017; Santoyo et al., 2017), that being a victim is a multifactorial phenomenon, so for its attention and prevention it must be a comprehensive program that includes social skills, self-control, motivation for academic activities, also supervising the establishment of positive interactions with school authorities (teachers, managers, etc.).

Thus, results suggest the need to increase the value of academic behavior by implementing stimulating intellectual activities that will decrease the relative value of coercive behavior.

### Social cognitive maps (social ecology)

To describe the stability of the behavior patterns of child victims (general objective) in relation to the social ecology of victims, bullies and matched controls, the findings from this study contrast with a rather large corpus of evidence which indicates that bullying is related to the absence of friendships (Eslea et al., 2003), lower levels of participation in social activities at school (Yüksel-Sahina, 2015), victims' isolation during activities (De Oliveira et al., 2016; Mendoza and Maldonado, 2017), and scant positive interaction with classmates (Mendoza and Maldonado, 2017). Based on the behavioral data and the socio-cognitive perspective (Farmer and Cairns, 1991), our results demonstrate that victims do not necessarily lack associations with their peers, since 66% of victims had three or more such links. This evidence is strengthened by the observation that, although victims are less responsive to their matched controls than the bullies, they do establish positive social interactions. It is important to point out that the methodological approach used to analyze the social ecology of the school setting (SCMs) has been recognized as a powerful predictor of social behavior, even more so than indirect psychometric measures (Santoyo et al., 2007a).

Another important result from this study is that bullies exhibit coercive behavior toward their classmates when there is a low probability that their attacks will be answered. This establishes a contingent relationship between the negative behavior and its consequences. Current findings (Santoyo et al., 2017) indicate that the violent behavioral pattern of some bullies is not regulated only by the relative reinforcement they receive, but also via negative reinforcement that victims receive (Sidman, 1989). Future studies should analyze the role of such regulatory mechanism in victims' behavior, incorporating the analysis of coercive interactions in the playground area with special emphasis on the analysis of the consequences received within the school environment that would provide valuable information, not only on the direct consequences of coercive episodes, but also on which participants receive by non-coercive behavior (Wilson and Herrnstein, 1985).

Finally, in this study the power imbalance or asymmetry between victim and bully was evaluated by analyzing coercive social interactions. Thus, we confirmed, as indicated by Atlas and Pepler (1998), that victims become involved in conflicts initiated by other classmates, and that bullies initiate such conflicts. This serves to demonstrate that—based on an observational methodology in natural settings—the coercive reciprocity model is considered a sensitive strategy for identifying bullies, and an option that permits a more complete description and understanding of the asymmetry that exists in the dyadic relationships between victims and bullies.

The empirical evidence provided in this study shows that social competency indexes, such as the Effectiveness, Responsiveness, and Reciprocity indexes, established between peers in the school context reflect functional mechanisms and facilitate a more complete description and explanation of the social relationships involved in the phenomenon of bullying in natural settings.

One limitation of the present investigation was not to include in the study the analysis of interactions in areas outside the classroom, such as the playground during games and recess activities, which can be suggested for future research.

## Ethics statement

This study was carried out in accordance with the recommendations of Mexican Society of Psychology, and ethics committee of Facultad de Psicología de la Universidad Nacional Autónoma de México, and National Council for Science and Technology, with written informed consent from school authorities in accordance to children' parents. The protocol was approved by Ethics Committee of Facultad de Psicología, Universidad Nacional Autónoma de México. Such resolution is supported by: Art. 8.03 (1) Ethical principles of psychologist and code of conduct. American Psychological Association, Effective January 1, 2017. 8. Research and publication, 8.03 (1).

## Author contributions

CS was the main researcher of this work; he provided de original idea and the organization of the research group. He organized the field work and the training of the research assistants; CS and BM wrote the first draft, by revising and rewriting the contents; Both CS and BM worked on the final draft and agrees to be accountable for all aspects of the work in ensuring that questions related to the accuracy or integrity of any part of the work are appropriately investigated and resolved.

### Conflict of interest statement

The authors declare that the research was conducted in the absence of any commercial or financial relationships that could be construed as a potential conflict of interest. The handling Editor declared a past co-authorship with one of the authors, CS, and a shared affiliation with one of the reviewers, ÁA.
